# A multi-image codebook approach for secure text transmission

**DOI:** 10.1371/journal.pone.0338836

**Published:** 2025-12-12

**Authors:** Omar Fitian Rashid, Saba A. Tuama, Humam Al-Shahwani

**Affiliations:** 1 Department of Geology, College of Science, University of Baghdad, Baghdad, Iraq; 2 Department of Engineering, University of Information Technology and Communications, Baghdad, Iraq; 3 Department of Computer Science, College of Science, University of Baghdad, Baghdad, Iraq; University of Anbar, IRAQ

## Abstract

In modern digital communication, Confidentiality of text transmission is remains a concern in the current online communication as cyber threats and intrusion. To address these challenges, this paper proposes a dual-layered security system that integrates cryptography and multi-image steganography to strengthen text protection during transmission. The cryptography layer is done based eight steps; in the first one, the message is converted to ASCII format, then convert the ASCII values into their equivalent binary numbers and make a complement to the binary values where each 0’s becomes 1’s and vice versa. In the next step, it needs to enter a key that includes a combination of characters, numbers, and special characters. This key is also converted to binary, and then the XOR operation is made between the message of the binary values and the key. In the fifth step, switching the values of each two adjacent binary values are together and converted to decimal values. While the second layer embeds the ciphertext in several cover images using a randomized codebook along with the Least Significant Bit (LSB) substitution, thus enhancing undetectability. Experimental evaluation demonstrates fast execution times for both the encryption/decryption processes and the multi-image hiding/extraction procedures. The achieved results validate that the proposed system provides an efficient and highly secure framework for protecting sensitive information.

## 1. Introduction

The security and confidentiality of information are becoming a critical matter. The transmission of information must be more secure because of the high number of threats that can access, alter, or steal this information. Therefore, a strong security system is needed to overcome these threats. Thus, the researcher’s goal is to develop a new, more secure encryption system. On the other hand, the information can be hidden within images, videos, etc., in order to secure its transmission over the internet and avoid intruders’ attention. Therefore, the combination of encryption and steganography is attracting more research attention [1].

Cryptography is applied in several types of research to encrypt and secure sending information. Pavithran et al. [2] proposed a novel cryptography technique by using DNA cryptography and finite automata theory. This system first generates a key to encrypt data, then generates a Mealy machine randomly to encode DNA sequences that achieve a secure ciphertext. Rudnytskyi et al. [3] suggested new asymmetric stream cipher construction by increasing both key and block sizes of encryption algorithms, which led to a rise in opportunities to improve cryptography systems. Al-Husainy et al. [4] implemented a lightweight cryptography system for IoT systems based on both substitution and transposition algorithms. This method depends on different block sizes that can be used with other IoT devices, and random keys can be generated by using DNA sequences. The achieved results showed that the suggested system can be used in different IoT devices with low memory needed and faster than other methods. Abu-Faraj and Alqadi [5] proposed a new method to enhance existing cryptography methods by using secret images to generate multiple private keys, and this led to an increase in the security level. Bermani et al. [6] presented a fast encryption technique based on the combination of Advanced Encryption Standard, Blowfish, and Message-Digest algorithm, where the proposed method achieved fast and robust data encryption. A new image encryption and authentication method is presented [7] based on the Elliptic Curve Diffie-Hellman with the use of 3D and 4D Arnold Cat maps, and it is applied for both grayscale and colour images. A new method is proposed using DNA encoding [8]. Then applying different matching algorithms to select the fastest one.

The steganography method can be defined as a method to store (hide) information or messages within text, images, or video to secure this information. Various new research were proposed in this field. Namasudra [9] built a novel cryptosystem for the cloud-based IoT infrastructure by using DNA cryptography and DNA steganography methods. This system encrypts data by using a long key and then hides these data inside the cover image, where this method also encrypts confidential data. A new steganography method is proposed by [10], which depends on Discrete Cosine Transform and Elliptic Curve Cryptography. This method led to enhanced steganographic image capacity by using the SegNet Deep Neural Network. Pramanik et al. [11] presented a two-layer security method, where the first layer is an encryption message based on the RSA algorithm, and the second layer is hiding achieved ciphertext in the cover image. Essa et al. [12] proposed a new steganography method based on a genetic algorithm, which is used to generate the keys that use selected secret positions. Jamel [13] proposed a new steganography method by using histogram modification methods to hide information where it is used to control the quality of steganography images. Hameed et al. [14] designed a new colour image steganographic technique by using a secret map. The proposed method uses a 3D map to hide the message within a selected part of an image to increase the complexity of breaking the system by the attackers. A new steganography method is presented by [15], where this method uses Krawtchouk-Tchebichef polynomials to select the area to hide the message within the cover image, and this method achieved high image quality. A new cryptography method is presented by [16] based on retina data. This is done by generating three keys. The first key is the distance between the center of the diagonals and the retina vessel’s end. In contrast, the second key is the distance between the center of the radius and the retina vessel’s end, and the third key is the diagonal-radius center and the retina vessel’s end. The current work aims to suggest a new text security method that includes both cryptography and steganography. Despite the availability of different encryption and steganography techniques, most existing systems are based on single-layer security schemes that are more vulnerable to the new methods of attack like statistical detecting, brute-force cryptography, and steganalysis. Furthermore, single image hiding greatly restricts distribution of payload which means that the embedded data would be vulnerable to alterations or deletion. Therefore, there has been an increasing demand to have a multi-layered security model which implements strong encryption with distributed steganographic embedding. The proposed system is motivated by this limitation and aims to provide a more robust, high-capacity and hard to detect secure method of communication by integrating dual-layer protection and multi-image codebook-based hiding.

Although many studies have explored hybrid cryptography–steganography systems, few have utilized the multi-image codebook selection together with multi-step binary transformation, to improve both confusion and diffusion of the concealed information. The majority of the existing methods also do not provide the flexibility of key control and are not distributed with embedding a series of cover images. To address these gaps, this study proposes a novel framework of text security, and the contributions of this study are as follows:

1. An efficient eight steps cryptography algorithm with binary complementing, XOR-based masking, switching and dual-key dependency.
2. Multi-image steganographic scheme with randomized codebook of distribution of ciphertext bits over two or three images to make it harder to decrypt and harder to detect statistically.
3. An implementation framework with all reproducibility documented hardware and software configuration.

## 2. Materials and methods

**Fig 1:**
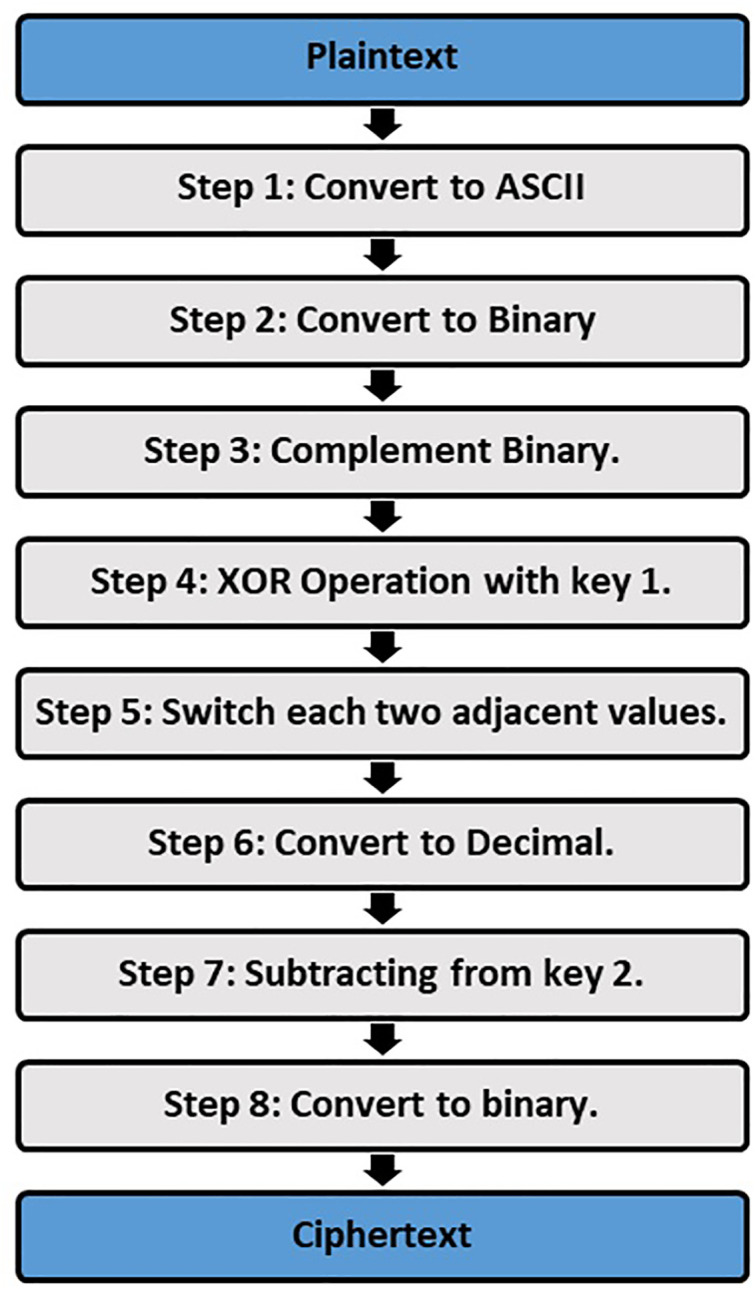
The first layer of protection with the applied Cryptography steps.

The proposed text security method is done based on two layers these are cryptography and steganography. In the first layer, Cryptography is used to encode text (plaintext) based on eight steps, and these steps are shown in Fig 1.

In the first step, plaintext characters are converted to their equivalent ASCII values; then, these ASCII values are converted to binary numbers. This is done by dividing the ASCII value by two and noting the remainder until you get to zero. The third step is done by making a complement for the binary values that were achieved from the previous step, where each 0’s is converted to 1’s, and vice versa. In the fourth step, an exclusive or (XOR) operation is used between binary values and the inserted key. The sender inserts this key, and the key needs to be converted to ASCII values, which are converted to binary values before executing the XOR operation. The fifth step is done by switching the values of each of the two adjacent binary values together, i.e., 01100010 becomes 10010001, and this operation is repeated for all the binary values. In the next step, the binary values are transformed to decimal numbers, and this operation is done by Multiplication; each binary digit by 2 raises to its position and sums up the results. The seventh step is done by subtracting each decimal value separately from the key, where these decimal values are achieved from the previous step.

In contrast, the key is a numerical value inserted by the sender. Finally, the last step of the cryptography operation is converting the final decimal values to binary values; these binary values are considered the final ciphertext that is used for the next protection layer. The first protection layer of the proposed method is cryptography, and its steps are illustrated in Algorithm 1.

Algorithm 1: Cryptography

**Input:** Plaintext, Key 1 (String), and Key 2 (Digital)

**Output:** Ciphertext

**1.Insert the plaintext, key 1 value, and key 2 value.**

**2.Convert plaintext to ASCII format:**

**For** i = 1 To Length (Plaintext)

x = Substring (plaintext, i, 1)

ASCII [i] = Asc (x)

**End for**

Len = i

**3.Convert ASCII to Binary format:**

**For** i = 1 To Length (Plaintext)

Number = Val (ASCII [i])

Binary_String = ““

**While** Number > 0

 Binary_String = Number Mod 2 & Binary_String

 Number = Number \ 2

**End while**

Binary [i] = Binary_String

**End For**

**4.Do Complement for all Binary values:**

**For** i = 1 To Length (Plaintext)

**For** j = 1 To 8

x = substring (Binary [i], j, 1)

**If** x = 0 Then

y = 1

**Else**

y = 0

**End If**

Comp_binary [i] = Comp_binary [i] & y

**End for**

**End for**

**5.Applied XOR operation between achieved binary values and key 1 value:**

i = 1

kc = 1

**While** i < Length (Plaintext)

**For** j = 1 To 8

k = substring (Comp_binary [i], j, 1) XOR substring (key1 (kc), j, 1)

xor_Result [i] = xor_Result [i] & k

**end for**

i = i + 1

**If** kc = Length (key) - 1 Then

kc = 1

Else

kc = kc + 1

**end If**

**end while**

**6.Switching the values of each two adjacent binary values:**

New_string = ““

**For** i = 1 To Length (Plaintext)

**For** j = 1 To 8 Step 2

**If** Substring (xor_Result [i], j, 1) = substring (xor_Result [i], j + 1, 1) Then

New_string = New_string & substring (xor_Result [i], j, 1) & substring (xor_Result [i], j + 1, 1)

**else**

New_string = New_string & substring (xor_Result [i], j + 1, 1) & substring (xor_Result [i], j, 1)

**end If**

**end for**

xor_Result [i] = New_string

**end for**

**7.Convert binary values to Decimal values:**

n = 0

**For** i = 1 To Length (string)

**For** s = 1 To 8

n = n + (substring (xor_Result [i], 8 - s + 1, 1) * (2 ^ (s - 1)))

**end for**

**If** n < 100 Then

n = 0 & n

**end If**

Decimal_numbers [i] = n

n = 0

**end for**

**8.Subtract each decimal value from key 2:**

**For** i = 1 To Length (string)

Decimal_numbers [i] = Decimal_numbers [i] - Key2

**end for**

**9.Convert Decimal to Binary format (Ciphertext).**

**For** i = 1 to Length (Plaintext)

Convert Decimal[i] to 8-bit Binary

Ciphertext = Ciphertext & Binary_String

**end for**

To ensure consistent security strength, Key 1 (character-based key) have a length of 8–32 characters which has enough randomness to perform XOR operations. Key 2 (the numeric key) is restricted to an integer range between 1–255 meaning that it can convert the result of the binary transformation to the operation of the 8-bit decimal operations. These parameters ensure that there is stability and there is no overflow in conversion.

**Fig 2:**
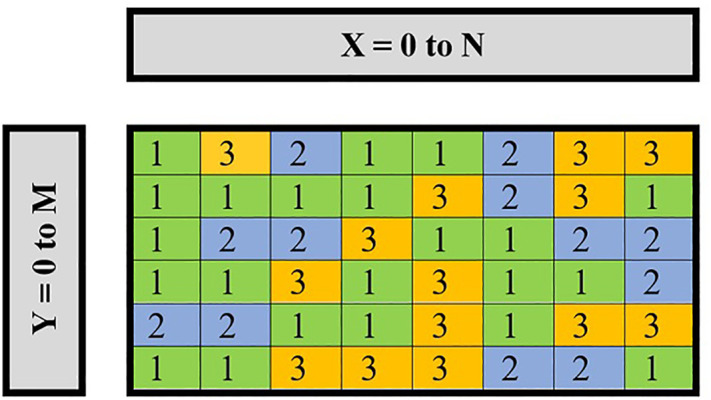
Generated codebook table used to select the appropriate cover image for each embedded bit.

**Fig 3:**
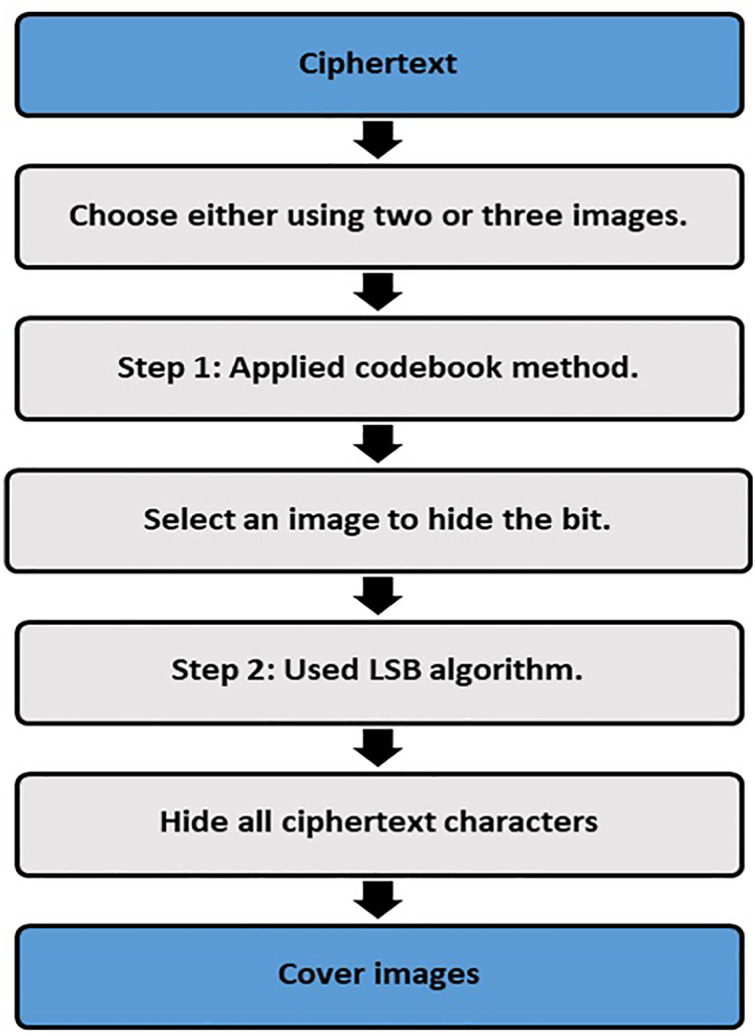
Steganography embedding process performed in the second protection layer.

In the second step, the steganography technique is used to hide binary values that were achieved in the previous step. This is done based on two methods, using two or three images to hide information inside it. These two methods are the codebook method and the least significant bit (LSB) method. The codebook is a document that is used for implementing code, and it contains a lookup table for coding and decoding. In the currently proposed method, a codebook table is built, as shown in Fig 2, in order to select which image to store the bit inside it, and the values inside the codebook table are generated randomly. The codebook has been generated based on image size, i.e., if the image size is 512x512, then the codebook size is also 512x512. For example, if the value inside the codebook table is 1, this means using image number one to hide the bit, and if the value is equal to 2, then the bit is stored inside image number two and so on for image three. After the application of method one and choosing the image, the LSB algorithm is applied, which involves changing the least significant bit of random pixel to store the binary data without noticeably affecting the image’s appearance. The applied Steganography steps are shown in Fig 3.

Fig 2 shows the codebook table with size NxM, and the values inside the table are generated randomly. The steganography process is done firstly by using either two or three images, then using a codebook table to decide which image is used. After that, the LSB algorithm is used to store bits inside random pixels from the image that is chosen by the codebook table. Finally, two or three cover images are sent to the receiver. The reasons why several cover images are used and a randomized codebook selection method is applied is to ensure the steganographic process is much more secure, robust and stealthy. Through the sharing of the ciphertext among two/three different pictures, the proposed system decreases the density of the embedding in each image, thus decreases detectability, and limits distortion in each cover picture. The second protection layer of the proposed method is steganography, and its steps are illustrated in Algorithm 2.

Algorithm 2: Steganography

**Input:** Ciphertext

**Output:** Cover images.

**1.Build codebook table:**

**For** i ← 1 to Height_of_image

**For** j ← 1 to Widtg_of_image

Codebook_table [i,j] = random (3)

**end for**

**end for**

**2.Hide ciphertext in cover images:**

Picture (0,0) = Length (ciphertext)

**for** i ← 1 to Length (ciphertext)

Bit = Substring (ciphertext, i, 1)

A = random ()

B = random ()

X= Codebook_table (A,B)

**If** X = 1 Then

Picture [A,B] = Picture1 [A,B] & Bit

**Else**

**If** X = 2 Then

Picture [A,B] = Picture2 [A,B] & Bit

**Else**

Picture [A,B] = Picture3 [A,B] & Bit

**end if**

**end if**

**end for**

**Fig 4:**
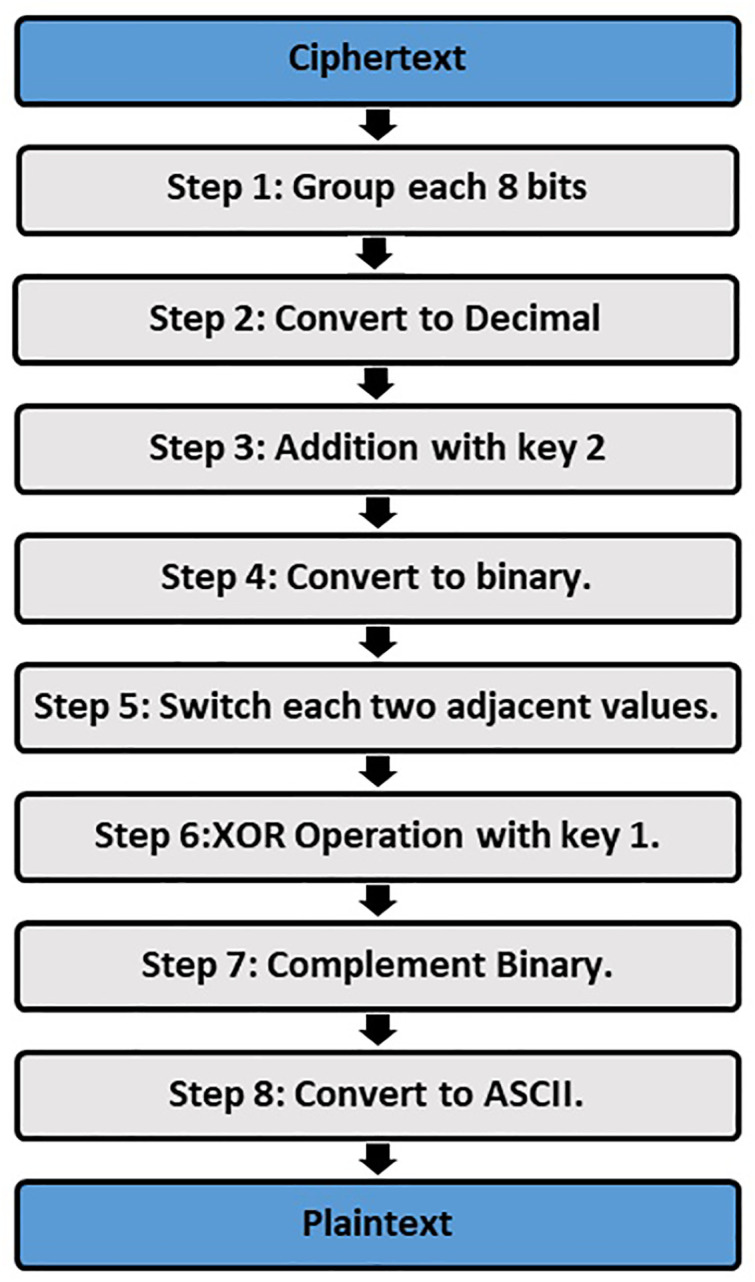
Extract the hidden bits inside images and apply the decryption process.

When the receiver receives the two or three cover images, he starts to extract the message (ciphertext) from the images by using the codebook table that the sender has generated. After that, he decoded the ciphertext to get the original message (plaintext). The process of decoding is done by inversing the coding method, as shown in Fig 4. The decoding process is done by:

- Using the two or three images that are sent from the sender.
- Using the codebook table.
- Extract the binary numbers that are hidden inside images.
- Group each 8 binary numbers and convert each group to its equivalent ASCII format. For example, 01010111 converts to 87.
- Applied addition operation between key 2 with the decimal numbers that were achieved from the previous step.
- Convert decimal numbers to binary format.
- Switch the values of each of the two adjacent binary values together.
- Applied XOR operation between binary values and key 1.
- Making a compliment for binary values achieved from the previous step.
- Convert the binary numbers to ASCII format.
- The last step is to convert ASCII format to characters, and these characters represent the sending message (plaintext).

## 3. Results and discussions

All the cryptography and steganography operations were performed on a computer with an Intel Core i7 CPU @ 3.6 GHz, 16 GB RAM, running Windows 10 (64-bit) to provide complete reproducibility of the experimental results. The implementation was written in R2022a of MATLAB/ Python 3.10 using Image Processing Toolbox. All the experiments were performed on uncompressed BMP images of 512 512 pixels. These specifications have now been added to clearly define the evaluation platform. In order to understand the steps of the proposed cryptography method to encode messages, the following example will explain and highlight these steps and their results. Suppose the plaintext is “ My new password is S2024-2025”

In the first step, the plaintext is converted to ASCII format:

77 121 32 110 101 119 32 112 97 115 115 119 111 114 100 32 105 115 32 83 50 48 50 52 45 50 48 50 53

The second step is done by converting the achieved ASCII to its equivalent Binary as follows:

01001101 01111001 00100000 01101110 01100101 01110111 00100000 01110000 01100001 01110011 01110011 01110111 01101111 01110010 01100100 00100000 01101001 01110011 00100000 01010011 00110010 00110000 00110010 00110100 00101101 00110010 00110000 00110010 00110101

In the third step, make a complement for Binary numbers:

10110010 10000110 11011111 10010001 10011010 10001000 11011111 10001111 10011110 10001100 10001100 10001000 10010000 10001101 10011011 11011111 10010110 10001100 11011111 10101100 11001101 11001111 11001101 11001011 11010010 11001101 11001111 11001101 11001010

Generate key one by converting it to binary numbers.

01000011 01110010 01111001 01110000 01110100 01101111 01100111 01110010 01100001 01110000 01101000 01111001 00100000 01101011 01100101 01111001 00100000 01101111 01101110 01100101

In the fourth step, the XOR operation is made between plaintext and key 1:

11110001 11110100 10100110 11100001 11101110 11100111 10111000 11111101 11111111 11111100 11100100 11110001 10110000 11100110 11111110 10100110 10110110 11100011 10110001 11001001 10001110 10111101 10110100 10111011 10100110 10100010 10101000 10111111 10101011

The fifth step is done by switching between each of the two adjacent values:

11110010 11111000 01011001 11010010 11011101 11011011 01110100 11111110 11111111 11111100 11011000 11110010 01110000 11011001 11111101 01011001 01111001 11010011 01110010 11000110 01001101 01111110 01111000 01110111 01011001 01010001 01010100 01111111 01010111

Then, convert binary values to their equivalent decimal numbers:

242 248 089 210 221 219 116 254 255 252 216 242 112 217 253 089 121 211 114 198 077 126 120 119 089 081 084 127 087

In the seventh step, made subtraction from decimal values by key 2:

201 207 48 169 180 178 75 213 214 211 175 201 71 176 212 48 80 170 73 157 36 85 79 78 48 40 43 86 46

In the last step, the decimal values are converted to Binary numbers, and this string of binary numbers will represent the ciphertext:

11001001 11001111 00110000 10101001 10110100 10110010 01001011 11010101 11010110 11010011 10101111 11001001 01000111 10110000 11010100 00110000 01010000 10101010 01001001 10011101 00100100 01010101 01001111 01001110 00110000 00101000 00101011 01010110 00101110

**Fig 5:**
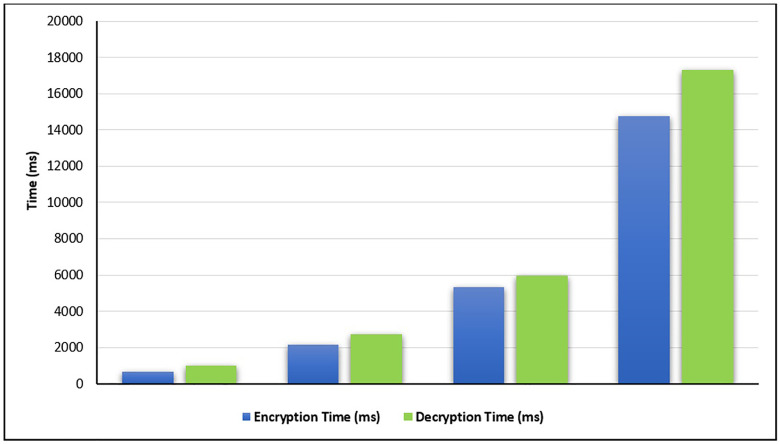
The cryptography time for different file sizes.

Two experiments are applied to evaluate the current proposed method: cryptography time and steganography time. For the first experiment, the cryptography time is calculated for different file sizes, and the calculating time is done in terms of milliseconds (ms), as stated in Table 1 and Fig 5.

**Table 1:** Encryption and Decryption Time (in terms of ms).

Size	Numbers of Characters	Encryption Time (ms)	Decryption Time (ms)
1k	1067	641	986
3K	3137	2130	2736
5K	5207	5344	5975
10K	10582	14781	17331

Tables 1 and Fig 5 demonstrate that both the encryption and decryption times of the proposed method are fast, where the encryption times for four different file sizes (1K, 3K, 5K, and 10K) are equal to 641 ms, 2130 ms, 5344 ms, and 14781 ms respectively. On the other hand, the decryption times for these files are equal to 986 ms, 2736 ms, 5975 ms, and 17331 ms respectively.

**Fig 6:**
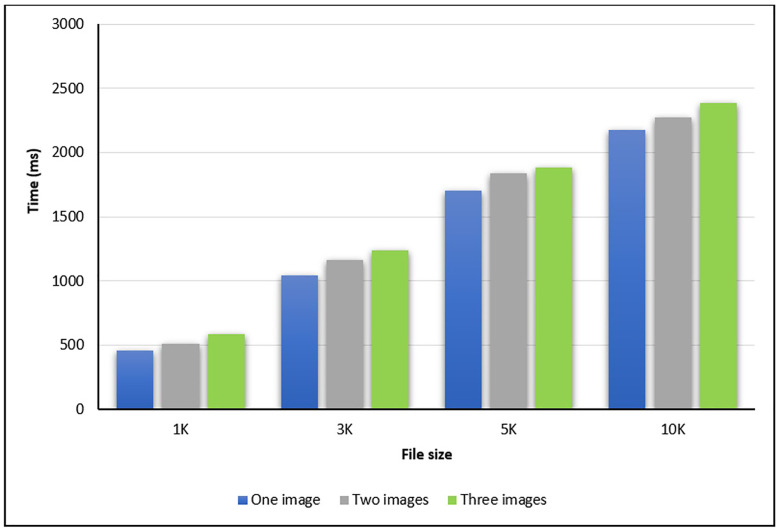
The total message hiding times for various sizes.

**Fig 7:**
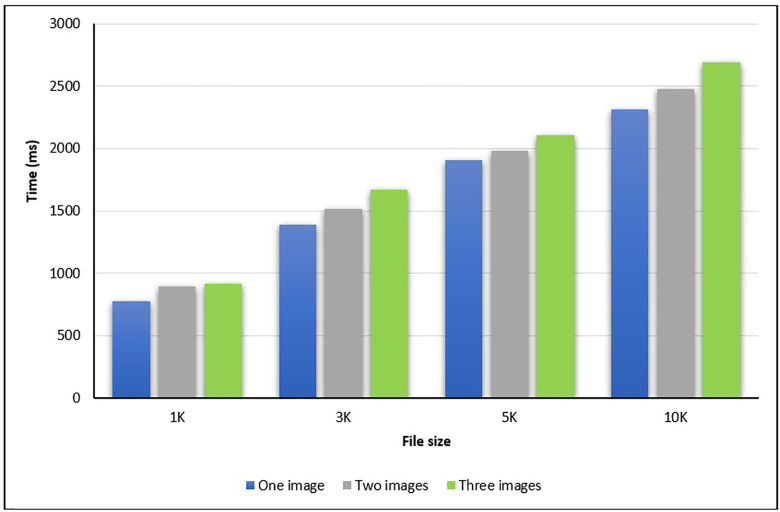
The total message extraction times for various sizes.

In the second experiment, the steganography time is calculated in milliseconds (ms) by using either one, two, or three images. The codebook is used to select which image to be stored the bit inside it. The steganography times for different file sizes are shown in Table 2, Fig 6, and Fig 7.

**Table 2:** Hiding and Extraction Time (in terms of ms).

Size	Time (ms)	Number of used images		
		One	Two	Three
1K	Hide message	459	513	588
	Extract message	773	893	917
3K	Hide message	1041	1165	1236
	Extract message	1390	1512	1668
5K	Hide message	1702	1839	1882
	Extract message	1906	1978	2103
10K	Hide message	2173	2274	2387
	Extract message	2310	2477	2691

As shown in Tables 2, Fig 6, and Fig 7, the times for both hiding and extraction messages are fast. The total time to hide the message within the covered image by using files with different sizes (1K,3K,5K, and 10K) and with either one, two, or three images showed that the time increased with increasing the number of the used images. The same results are achieved for the extracting times for different numbers of images.

## 4. Conclusions

A new security system is proposed by combining both cryptography and steganography techniques. The cryptography phase includes eight steps to encode the sending text, starting by converting the message to ASCII format and then to binary numbers. After that, a complement operation is applied. Then, the XOR operation is executed between binary values and key, and the values of each two adjacent binary values are switched together. These values are converted to decimal values, and these values are subtracted from the key value. The last step is converting decimal values to their equivalent binary values that represent the achieved ciphertext. In the steganography phase, the achieved ciphertext is hiding within one, two, or three images. This method is done by building a codebook table and using the LSB method to hide the bits. The performance evaluation of the proposed system showed that the time obtained for executing the tests by the proposed method is faster than previously published methods. For future work, the inclusion of adaptive deep learning models to produce generating dynamic codebooks and optimizing key selection, using convolutional neural networks to perform automated image selection to enhance it further against steganalysis, and higher-capacity embedding schemes to transmit large volumes of encrypted text. Additionally, the extension of the system to accommodate video based multi-frame hiding and real-time secure communication is a promising extension.
